# Host-derived stimuli alter intracellular calcium levels in *Pseudomonas aeruginosa,* revealing its potential role as a second messenger

**DOI:** 10.1128/aem.01359-26

**Published:** 2026-08-28

**Authors:** Aya Kubo, Reygan E. Braga, Dulandika Malalage, Maha M. Achour, Marianna A. Patrauchan

**Affiliations:** 1 Department of Microbiology and Molecular Genetics, Oklahoma State University 7618, Stillwater, Oklahoma, USA; The University of Tennessee Knoxville, Knoxville, Tennessee, USA

**Keywords:** R-GECO1 probe, cycloserine, calcium signaling, intracellular pH, quorum sensing, asparagine, ATP, ADP, AMP, PQS, HOCl

## Abstract

Calcium signaling is a fundamental cellular communication mechanism, well established in eukaryotes, that relies on calcium ions (Ca^2+^) acting both as first and second messengers to regulate diverse life-sustaining processes. In bacteria, although the significance of Ca^2+^ regulation is recognized, it is commonly attributed to its role as a first messenger, while the mechanistic basis for its capacity as a second messenger remains elusive. Previously, we have shown that *Pseudomonas aeruginosa,* an opportunistic pathogen causing life-threatening infections in humans, possesses the key prerequisites for intracellular Ca^2+^ (Ca^2+^_in_) signaling, including tightly controlled Ca^2+^_in_ homeostasis, transient changes in Ca^2+^_in_ levels in response to exogenous Ca^2+^, and Ca^2+^-regulated gene expression. In addition, we and others identified several key components of the Ca^2+^ signaling network*,* including Ca^2+^ sensors, transporters, and regulators. The remaining question is whether the levels of Ca^2+^_in_ alter in response to any extracellular stimuli other than Ca^2+^. To determine this, we have used a cytoplasmic Ca^2+^ probe, R-GECO1, that produces a Ca^2+^-dependent fluorescence and tested a variety of extracellular stimuli, including ions, monosaccharides, amino acids, nucleotides, quorum-sensing signaling molecules, reactive oxygen species, and antibiotics. We observed changes in Ca^2+^_in_ levels in response to several molecules, particularly glucose, asparagine, ATP, ADP, AMP, HOCl, and cycloserine. The results provide the first experimental evidence that, like eukaryotes, *P. aeruginosa* responds to select, chemically diverse host-related stimuli by altering its Ca^2+^_in_ levels, supporting a potential signaling role of Ca²^+^_in_ in bacteria.

**IMPORTANCE:**

Cells use signaling to communicate and respond to their environment. Although the role of calcium ions (Ca²^+^) as a signaling molecule, regulating multiple essential processes, has been well established in eukaryotes, Ca²^+^ signaling in bacteria remains understudied. Previous research has indicated the role of Ca²^+^ in bacterial regulation as a first messenger; however, its role as a second messenger is not yet recognized. Here, we show, for the first time, that *P. aeruginosa*, an important human pathogen, alters its intracellular Ca²^+^ levels in response to various external stimuli commonly associated with the host environment. These findings provide the mechanistic basis for *P. aeruginosa* to use Ca²^+^ as a second messenger. Understanding these Ca^2+^ responses provides novel insights into Ca^2+^-mediated adaptation of invading pathogens to the host environment and may inform the development of new antimicrobial strategies.

## INTRODUCTION

To survive in constantly changing environments, cells rely on signaling. This process requires two types of messengers: first messengers, which originate outside the cell and, upon binding to cell surface receptors, trigger a cellular response; and second messengers, whose intracellular levels change in response to extracellular signals, transducing them into cellular responses. Among them, calcium ions (Ca^2+^) can transmit information as both first (extracellular) messenger and second (intracellular) messenger (1). In eukaryotes, intracellular Ca^2+^ (Ca^2+^_in_) signaling is well established and relies on spatial and temporal changes in the cytosolic concentration of Ca^2+^ from 10^−7^ M in the resting state to 10^−5^ M in the excited state in response to various extracellular and intracellular stimuli (2). These transient fluctuations are coordinated by Ca^2+^ transporters and Ca^2+^ buffering systems, and their frequency and amplitude define a signal that is recognized and decoded by Ca^2+^-binding proteins (CaBPs) to form cellular responses (3–5). Ca^2+^_in_ signaling is involved in almost every aspect of eukaryotic cell life and death by regulating a large number of essential processes, ranging from cellular division and apoptosis to immune responses and defenses against bacterial infection (reviewed in references 1, 6). However, the role of Ca^2+^ regulation and signaling in bacteria remains underappreciated.

Work by colleagues and us showed that in bacteria, Ca^2+^ plays a regulatory role at the levels of gene expression and protein activity, controlling a variety of physiological processes, such as biofilm development, spore formation, chemotaxis, transport, and virulence (7–9). Most of our work on Ca^2+^ signaling has focused on *Pseudomonas aeruginosa,* a major human pathogen causing life-threatening acute and chronic respiratory, wound, and other infections (10). *P. aeruginosa* is particularly well known for infecting the airways of cystic fibrosis (CF) patients, leading to fatal obstructive lung disease (11–13). The success of *P. aeruginosa* as a pathogen is thought to be due in part to its physiological plasticity, enabled by potent signaling systems that control adaptive changes and resistance (reviewed in references 14, 15). As a result of CF, the extracellular concentrations of Ca^2+^ can reach up to 5 mM in the nasal secretions and saliva (16–18). Our previous studies showed that these levels of Ca^2+^ have a significant regulatory impact on various cellular processes in *P. aeruginosa* (19–23). We have observed positive regulation of pyocyanin, secreted proteases, alginate, pyoverdine, and pyochelin (23–27), enhancing *P. aeruginosa* pathogenicity during chronic infections and resistance (20, 23, 28, 29), as well as negative regulation of the type 3 secretion system and its effectors, major determinants of *P. aeruginosa* ability to cause acute infections (30). These findings suggest that sensing elevated Ca^2+^ levels within the nasal passages, where *P. aeruginosa* resides during the early stages of infection, triggers adaptive changes (31–33) that facilitate host colonization.

To begin establishing the molecular mechanisms of Ca^2+^_in_ signaling in *P. aeruginosa*, we monitored the Ca^2+^_in_ levels in cells exposed to various extracellular Ca^2+^ (Ca^2+^_ex_) concentrations by using the Ca^2+^-binding luminescent reporter protein aequorin (34). We determined that *P. aeruginosa* maintains a low micromolar level of Ca^2+^_in_ under resting conditions (no Ca^2+^ added), which, in response to 1 mM Ca^2+^_ex_, transiently increases and then, in spite of the steep gradient, returns to the resting level within minutes (34). In addition, we and others identified several key components of the Ca^2+^ signaling network*,* including Ca^2+^ sensors, the histidine kinase LadS (35, 36) and the EF-hand protein EfhP (19, 28, 37), the two-component regulatory system CarRS/BqsRS (20, 38), and transporters (34) that mediate Ca^2+^ regulation of gene expression or control Ca^2+^ cellular homeostasis. Taken together, tightly controlled Ca^2+^_in_ homeostasis, transient changes in Ca^2+^_in_ in response to exogenous Ca^2+^, and Ca^2+^-regulated gene expression satisfy the key prerequisites for Ca^2+^_in_ signaling as established in eukaryotes. A remaining unanswered question is whether Ca^2+^_in_ responds to extracellular stimuli other than Ca^2+^, which would provide the basis for Ca^2+^_in_ to function as a second messenger. Although limited studies have reported Ca^2+^_in_ fluctuations in response to bacterial metabolites, such as butane 2,3-diol in *E. coli* (39), flavonoid or isoflavone in *Rhizobium leguminosarum* (40), and graphene oxide in *B. subtilis* (41), no systematic studies have addressed host-related factors triggering Ca^2+^_in_ response. Furthermore, no such studies have been reported for *P. aeruginosa*.

Although aequorin has been widely used to measure Ca^2+^_in_ levels, the need for its reconstitution with the chromophore coelenterazine (34) limits applications. Here, we applied a fluorescent probe from the family of Genetically Encoded Calcium Indicators (GECI), R-GECO1, to monitor Ca^2+^_in_ dynamics in live *P. aeruginosa* cells in response to nutritional, signaling, and stress-related stimuli. The R-GECO1 reporter has been developed for specific detection and monitoring of cellular Ca^2+^ in eukaryotic cells (42, 43). It is composed of a Ca^2+^-binding calmodulin (CaM) domain, a circularly permuted red fluorescent protein (cpRFP, mApple lineage), and a CaM-binding M13 peptide. When the CaM domain binds Ca^2+^ (Kd ~0.48 µM), it undergoes a conformational change promoting interactions with the M13 peptide. This, in turn, alters the chromophore environment of cpRFP, emitting red fluorescence with the intensity proportional to Ca²^+^_in_ levels (44). As Ca²^+^_in_ levels fall back, Ca²^+^ dissociates from CaM, quickly reducing the fluorescence (decay rate of ~0.120 s [45]). Thus, R-GECO1 dynamically tracks cytoplasmic Ca²^+^ levels, enabling real-time, noninvasive monitoring. Here, we relate the intensity of the measured R-GECO1 fluorescence to changes in Ca^2+^_in_ levels. Using this probe, we determined that *P. aeruginosa* Ca^2+^_in_ levels change in response to selected, chemically diverse stimuli, providing evidence for a functional Ca^2+^_in_ signaling system capable of recognizing multiple environmental cues. These findings lay the groundwork for the next goal of identifying the molecular mechanisms that transduce Ca^2+^_in_ signals to form cellular responses.

## RESULTS

### Construction and evaluation of R-GECO1 and GCaMP6f indicators for monitoring Ca^2+^_in_ in *P. aeruginosa*

**Fig 1:**
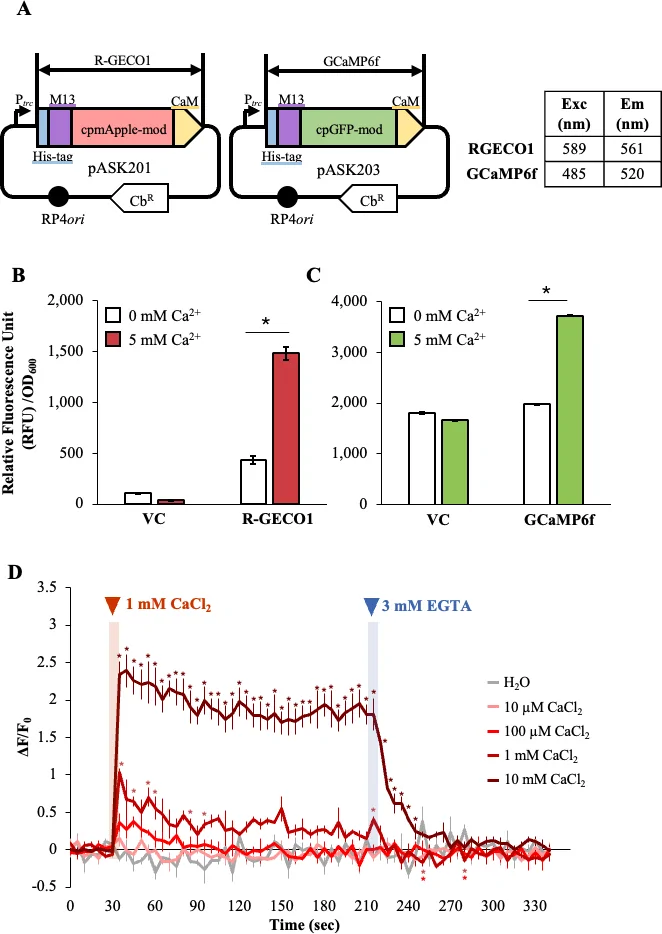
Construction and evaluation of R-GECO1 and GCaMP6f indicators for monitoring Ca^2+^_in_ levels. (**A**) Construction of genetically encoded calcium indicators (in) R-GECO1 and GCaMP6f for expression in PAO1. (**B**) The relative fluorescence intensity in PAO1 carrying pMF36 (VC), pASK201 (R-GECO1), or (**C**) pMF36 (VC) and pASK202 (GCaMP6f). Cells were grown to mid-log phase in biofilm minimal medium (BMM) with or without 5 mM Ca^2+^. (**D**) The relative fluorescence intensity of R-GECO1 (Δ*F*/*F*_0_) in mid-log PAO1 carrying pASK201, calculated as Δ*F* = RFU at each time point − *F*_0_, where *F*_0_ = average basal RFU before injection. The resulting Δ*F* was normalized by *F*_0_. Water (negative control) or 10 µM to 10 mM CaCl_2_ and 3 mM EGTA were added at the time indicated by the arrow. The average and the standard error values obtained from three to four biological replicates are shown. For statistical analysis, Student’s *t*-test (B and C) or a one-way ANOVA with Dunnett’s post hoc statistical analysis (D) (*, *p* < 0.05) was performed.

To assess the utility of GECIs for tracking Ca^2+^_in_ dynamics in *P. aeruginosa* PAO1, we first constructed two plasmids based on the broad-host-range vector pMF36 (46), each carrying a fluorescent probe under the constitutive *trc* promoter (Fig. 1A): R-GECO1 (red [43]) and GCaMP6f (green [47]). The resulting plasmids, pASK201 and pASK203, were each transformed into PAO1. R-GECO1 contains a derivative of mApple, whereas GCaMP6f contains a derivative of EGFP (43, 48). To evaluate their performance against *P. aeruginosa,* fluorescence intensity was measured in exponential-phase PAO1 cells grown with or without 5 mM Ca^2+^ (Fig. 1B and C). Both indicators were functional and exhibited significantly higher fluorescence in Ca^2+^-supplemented conditions. However, due to the higher background from the intrinsic green fluorescence of *P. aeruginosa,* R-GECO1 was chosen for the following studies.

In previous work, using aequorin as a Ca^2+^-reporter, we measured Ca^2+^_in_ in *P. aeruginosa* and found that its concentration transiently increases upon exposure to 1 mM Ca^2+^_ex_ (34). To evaluate whether R-GECO1 detects changes in Ca^2+^_in_ levels in response to increasing concentrations of Ca^2+^_ex_, cells grown without supplemental Ca^2+^ were challenged with Ca^2+^_ex_ ranging from 10 μM to 10 mM Ca^2+^, as described in Materials and Methods (Fig. 1D). These concentrations reflect the range of Ca^2+^ levels commonly present in the natural environments, where *P. aeruginosa* resides (49) or nasal secretions and saliva of CF patients (50), where it undergoes the early stages of pathoadaptation (31–33). Exposure to Ca^2+^_ex_ triggered a rapid increase in R-GECO1 fluorescence normalized by OD_600_ reflecting the increase in Ca^2+^_in_ levels within 4 s, with the peak fluorescence intensity proportional to the concentration of Ca^2+^ added (Fig. 1D). Specifically, exposure to 1 mM and 10 mM Ca^2+^ resulted in 2.5- and 12-fold increases in normalized fluorescence, respectively, compared with the H_2_O control. As expected, subsequent treatment with EGTA restored the fluorescence levels to baseline (Fig. 1D). These results show that, as established in reference 44, R-GECO1 reliably emits red fluorescence with intensity proportional to the Ca²^+^_in_ level, as previously characterized using aequorin (34).

**Fig 2:**
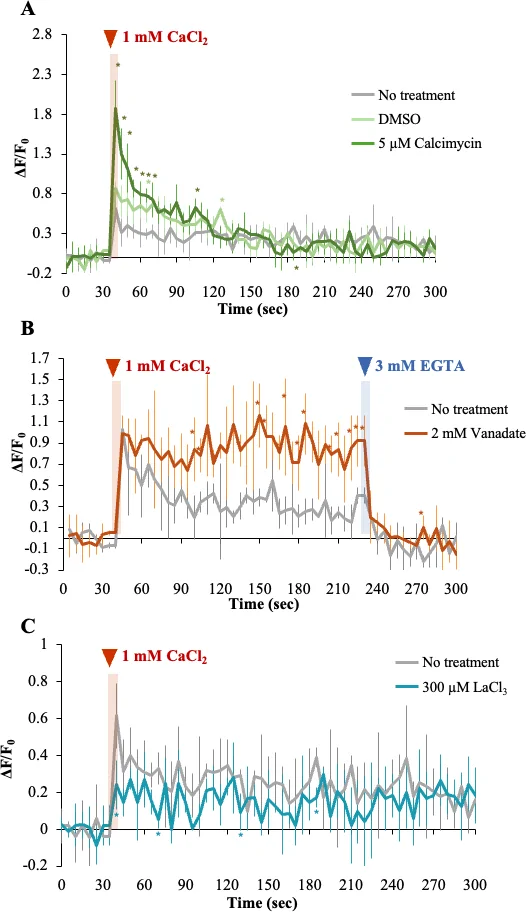
Validating R-GECO1 indicator for monitoring Ca^2+^_in_ levels. The relative fluorescence intensity of R-GECO1 (Δ*F*/*F*_0_) in PAO1 carrying pASK201 was calculated as Δ*F* = RFU at each time point − *F*_0_, where *F*_0_ = averaged basal RFU before injection. The resulting Δ*F* was normalized by *F*_0_. The culture was treated with 5 µM calcimycin (**A**), 2 mM vanadate (**B**), or 300 µM lanthanum chloride (**C**), and then exposed to 1 mM CaCl_2_ and 3 mM EDTA at the time indicated by the corresponding arrows. The average and the standard error values obtained from three to four biological replicates are shown. For statistical analysis, a one-way ANOVA with Dunnett’s post hoc statistical analysis (*, *p* < 0.05) was performed.

Although CaM, defining Ca^2+^ sensitivity of R-GECO1, has a much higher affinity for Ca^2+^ (Kd 3 × 10⁻⁶ to 2 × 10⁻⁵ M) (51) than for Mg^2+^ (Kd 10^−4^ to 10^−2^ M) (52), we aimed to confirm that R-GECO1 specifically reports Ca^2+^_in_ and not Mg^2+^ in *P. aeruginosa*. For this, PAO1 cells grown in the absence of Ca^2+^ were exposed to Mg^2+^ by adding 0.01–10 mM MgCl_2_. As expected, we observed no increase in R-GECO1 fluorescence (see Fig. S1 at https://doi.org/10.5281/zenodo.22032718). To further verify that R-GECO1 reflects changes in Ca^2+^_in_, PAO1 cells were treated with calcimycin, a Ca^2+^ ionophore known to facilitate Ca^2+^ entry into cells (53), and subsequently exposed to 1 mM Ca^2+^ (Fig. 2A). Given that calcimycin was dissolved in DMSO, an equivalent concentration of DMSO was included as a control. Treatment with 5 μM calcimycin led to a threefold increase in fluorescence in response to Ca^2+^_ex_ relative to untreated cells and a twofold increase compared to DMSO-treated cells (Fig. 2A). These results further support that R-GECO1 can be used for real-time monitoring of Ca^2+^_in_ fluctuations within the cytoplasm of PAO1 cells. Below, we report normalized R-GECO1 fluorescence as reflecting changes in Ca^2+^_in_ levels.

### Ca^2+^_in_ homeostasis in *P. aeruginosa* requires functional Ca^2+^ channels and ATPases

To further validate the utility of R-GECO1 and expand our previous findings that Ca^2+^ channels and ATPases maintain Ca^2+^_in_ homeostasis (34), we examined the effects of two inhibitors: vanadate (54, 55) and LaCl_3_ (56, 57) on Ca^2+^_in_ responses to Ca^2+^_ex_. For this, PAO1 cells were pretreated with the inhibitors, then challenged with 1 mM Ca^2+^, and R-GECO1 fluorescence was monitored. Vanadate, a known inhibitor of P-type and ABC-type ATPases (54, 55), is expected to block ATPase-mediated Ca^2+^ efflux. Consistent with this, treatment with 2 mM vanadate did not affect the initial rapid increase in Ca^2+^_in_ upon Ca^2+^_ex_ addition but prevented the subsequent return to basal levels (Fig. 2B). Treated cells maintained Ca^2+^_in_ levels at more than threefold higher than the baseline, which was restored only upon the addition of 3 mM EGTA. LaCl_3_, a well-established Ca^2+^ antagonist, blocks Ca^2+^ channels and may also interfere with Ca^2+^-binding ATPases (56, 57). As expected, pretreatment with 300 μM LaCl_3_ significantly inhibited Ca^2+^ uptake, resulting in a threefold reduction in the increase of Ca^2+^_in_ following Ca^2+^_ex_ addition (Fig. 2C). Collectively, these results support the conclusion that *P. aeruginosa* employs multiple transport systems to maintain Ca^2+^ homeostasis and further validate the utility of R-GECO1 for real-time monitoring of Ca^2+^_in_ dynamics in live bacterial cells.

### The intracellular pH responds to exogenous Ca^2+^

**Fig 3:**
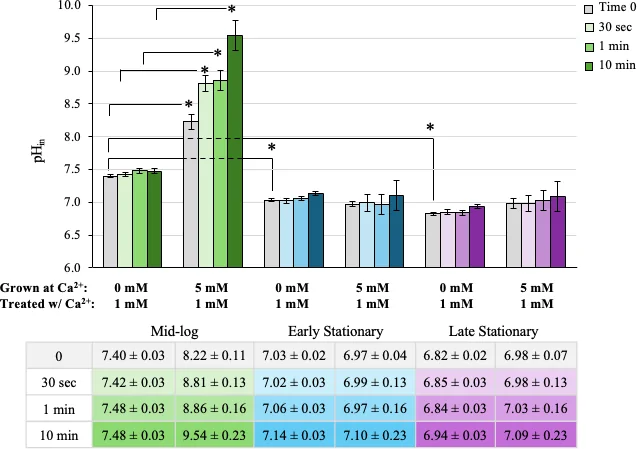
The effect of Ca^2+^_ex_ on intracellular pH. Mid-log (12 h), early stationary (24 h), or late stationary (36 h) phase PAO1 cells grown in BMM with or without 5 mM Ca^2+^ were challenged with 1 mM Ca^2+^. The intracellular pH (pH_in_) was monitored before (0) and after 30 s, 1 min, and 10 min of exposure to Ca^2+^. The average and standard errors (*n* = 4) are shown. One-way ANOVA with Tukey-Kramer post hoc statistical analysis (*, *p* < 0.05) was performed.

Given that pH can impact the chromophore conformation in red fluorescence proteins (58), we assessed whether changes in intracellular pH (pH_in_) occur in response to Ca^2+^_ex_ exposure. For this purpose, a pH indicator, PHP (59), was expressed in *P. aeruginosa* PAO1 to monitor pH_in_. First, a calibration curve was generated using mid-log phase PAO1 cells expressing PHP (see Fig. S2B and C at https://doi.org/10.5281/zenodo.22032718), which was used to quantify pH_in_ changes in PAO1 upon exposure to 1 mM Ca^2+^. For this, cells were grown in the presence or absence of 5 mM Ca^2+^ to mid-log, stationary, or late stationary phases of growth. These cells were collected and treated with 1 mM Ca^2+^ for 30 s, 1 min, and 10 min. Prior to and following these treatments, their pH_in_ was measured. We observed that mid-log cells grown in 5 mM Ca^2+^ had higher pH_in_ (8.22) than mid-log cells grown at no Ca^2+^ (pH_in_ 7.4) (time 0, shown in gray) (Fig. 3). No significant changes in pH_in_ were observed in stationary-phase (early or late) cells grown at 5 mM Ca^2+^ vs their counterparts grown at no Ca^2+^, likely due to a stronger control of pH_in_ homeostasis during stationary phase (60), which was possibly enhanced by Ca^2+^. When comparing pH_in_ in stationary-phase (early and late) and mid-log cells grown at no Ca^2+^ (time 0, in gray), we observed a general trend toward lower pH_in_ from 7.4 in mid-log to 7.03 in early stationary and 6.82 in late stationary. In response to 1 mM Ca^2+^_ex_ exposure for 30 s, 1 min, and 10 min, pH_in_ remained stable in mid-log cells grown without Ca^2+^ but gradually increased from 8.22 to 9.54 within 10 min in mid-log cells grown at 5 mM Ca^2+^ (Fig. 3, shown in light, medium-light, and dark green). In contrast, no significant difference in pH_in_ was observed in stationary-phase cells grown at either Ca^2+^ condition upon up to 10 min of Ca^2+^ exposure (Fig. 3, shown in blue and purple). These results indicate that (1) challenging mid-log cells pre-grown in Ca^2+^ with additional Ca^2+^ increases their pH_in_, with a further rise upon longer exposure (2); pH_in_ in stationary-phase cells is slightly lower than in mid-log cells and does not respond to the addition of Ca^2+^. To prevent potential interference of changing pH_in_ on R-GECO1 fluorescence, we proceeded to use stationary-phase PAO1 cells.

### *P. aeruginosa* alters Ca^2+^_in_ levels in response to distinct external stimuli

Together with our previous findings (34), the results presented so far confirm that Ca^2+^_in_ homeostasis is tightly regulated in *P. aeruginosa*. However, in order for Ca^2+^_in_ to function as a second messenger and support functional Ca^2+^_in_ signaling, as established in eukaryotes, the levels of Ca^2+^_in_ have to respond to external stimuli. To investigate this in *P. aeruginosa,* we monitored Ca^2+^_in_ dynamics in cells exposed to a range of physiologically and clinically relevant molecules. Among these, we tested several types of molecules that *P. aeruginosa* commonly encounters or produces during infection in the host, including ions, monosaccharides, amino acids, signaling, and oxidative stress-causing molecules, as well as antibiotics used to treat *P. aeruginosa* infections. To ensure adequate Ca^2+^ availability and clinical relevance, *P. aeruginosa* was grown in medium supplemented with 5 mM Ca^2+^.

#### Ions

Ion imbalance is a hallmark of CF, where NaCl concentration is commonly elevated in the airway fluids (61) and has a significant impact on osmoregulation and virulence of *P. aeruginosa* (62–64). To examine a possible impact of NaCl on Ca^2+^_in_, *P. aeruginosa* cells carrying R-GECO1 were challenged with 0.245 mM–1 M NaCl. No effect was observed at 0.245–200 mM NaCl, whereas a significant increase in Ca^2+^_in_ levels was detected at 1 M NaCl (see Fig. S4A at https://doi.org/10.5281/zenodo.22032718). Because 1 M NaCl induces osmotic shock in *P. aeruginosa* (64)*,* causing envelope stress and membrane perturbation, this may facilitate Ca^2+^ entry and disturb Ca^2+^_in_ homeostasis, resulting in elevated Ca^2+^_in_ levels. Consistent with this possibility, we detected increased R-GECO1 fluorescence in PAO1 supernatants following treatment with 1 M NaCl compared to saline control, supporting the conclusion that this treatment is severe enough to cause membrane damage (see Fig. S4B at https://doi.org/10.5281/zenodo.22032718). Since R-GECO1 fluorescence intensity was similar in both the water control and isotonic saline sample (145 mM NaCl), the latter was used as a diluent to provide osmotic balance for the rest of the study.

**Fig 4:**
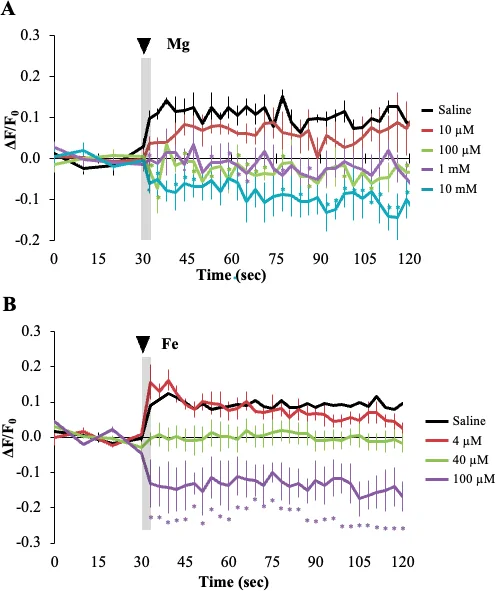
The effect of ions on Ca^2+^_in_ levels. Relative fluorescence change (Δ*F*/*F*_0_) of R-GECO1 in stationary-phase PAO1 grown in BMM supplemented with 5 mM Ca^2+^. Relative fluorescence intensity (Δ*F*/*F*_0_) was calculated as Δ*F* = RFU at each time point − *F*_0_, where *F*_0_ = averaged basal RFU before injection. The resulting Δ*F* was then normalized by *F*_0_. MgCl_2_ (**A**) or FeSO_4_ (**B**) was added at the time indicated by the arrows, and R-GECO1 fluorescence was measured. The average and the standard error values obtained from three to four biological replicates are shown. A one-way ANOVA with Dunnett’s post hoc statistical analysis (*, *p* < 0.05) was performed.

Considering that the supply of exogenous magnesium and iron is essential for bacterial survival (reviewed in reference 65), we tested whether these ions have an impact on Ca^2+^_in_ homeostasis. Exposure to 10 μM–10 mM MgCl_2_ led to a dose-dependent decrease in Ca^2+^_in_ becoming statistically significant at 100 μM (Fig. 4A). Similarly, the addition of 40 μM–0.1 mM FeSO_4_ also induced a dose-dependent reduction in Ca^2+^_in_ with statistically significant impact at 100 μM (Fig. 4B). Because Fe^2+^ oxidizes to Fe^3+^ under oxic conditions, we assessed the level of Fe^2+^ at the end of the experiment and found that at least 70% of the initially added Fe^2+^ remained. Together with the rapid decrease in Ca^2+^_in_ following FeSO_4_ addition (Fig. 4B), this suggests that the detected Ca^2+^_in_ response is attributable to Fe^2+^. Given the very low affinity of Mg and Fe for CaM (66) and the verified lack of R-GECO1 fluorescence in response to Mg (see Fig. S1 at https://doi.org/10.5281/zenodo.22032718), these effects are likely not due to CaM conformational interference, though their potential in quenching fluorescence (67) cannot be excluded. Furthermore, to determine whether the reduction in R-GECO1 fluorescence was due to changes in pH_in_, we measured pH_in_ in stationary-phase cells exposed to 1 mM FeSO_4_ or 10 mM MgCl_2_ and observed no significant differences (see Fig. S3 at https://doi.org/10.5281/zenodo.22032718). These results indicate that Mg and Fe may activate Ca^2+^ efflux. It is also possible that the Ca^2+^_in_ decrease in response to Mg and Fe sets a regulatory signal that coordinates the uptake of these essential metals.

#### Sugars

**Fig 5:**
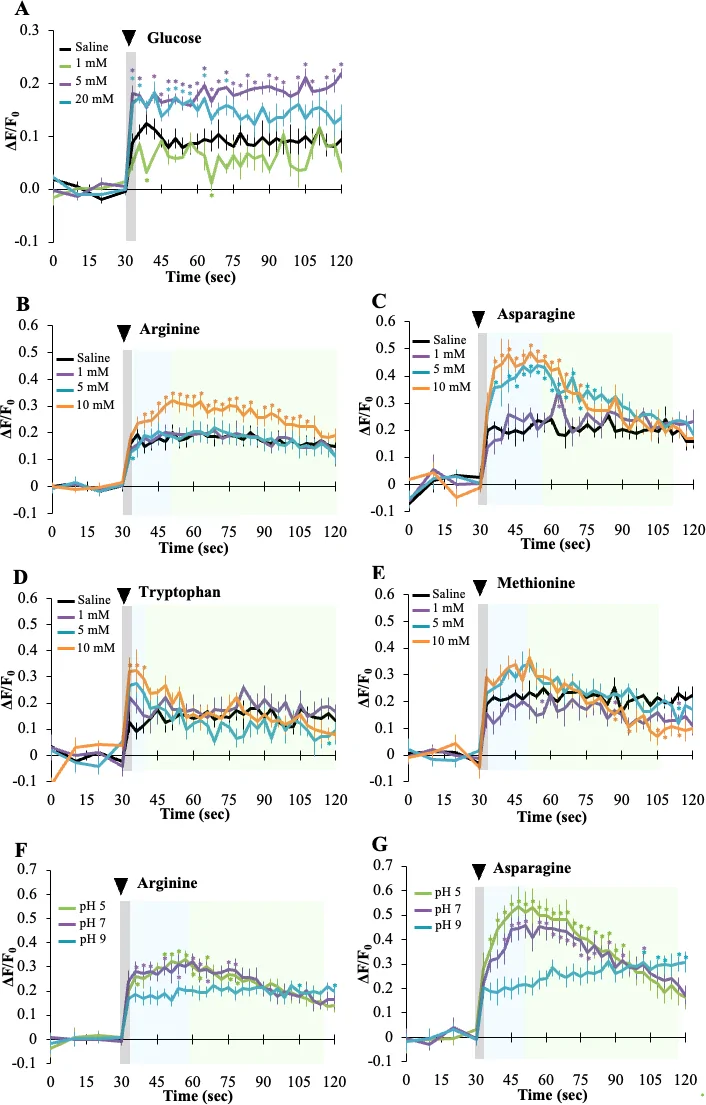
The effect of glucose and amino acids on Ca^2+^_in_ levels. The relative fluorescence intensity of R-GECO1 (Δ*F*/*F*_0_) in stationary-phase PAO1 grown in BMM with 5 mM Ca^2+^ was calculated as Δ*F* = RFU at each time point − *F*_0_, where *F*_0_ = average basal RFU before injection. The resulting Δ*F* was normalized by *F*_0_. Glucose (**A**), Arg (**B**), Asn (**C**), Trp (**D**), or Met (**E**) at pH 6.8–7.0 was added at the time indicated by the arrows, and R-GECO1 fluorescence was measured. To elucidate the pH effect, 5 mM Arg (**F**) or Asn (**G**) at pH 5–9 was added at the time indicated by the arrows. Statistical analysis was done for comparisons at pH 5 and 9 to pH 7. The average relative fluorescence intensity and the standard error values obtained from three to four biological replicates are shown. A one-way ANOVA with Dunnett’s post hoc statistical analysis (*, *p* < 0.05) was performed.

Glucose and fucose were selected for analysis because of their roles in host immune responses and their use as adjunct therapies for reducing bacterial attachment (68–71), with glucose but not fucose serving as a major carbon source. We tested a concentration range of 1–20 mM, which is commonly used in growth studies (72, 73). Although no significant Ca^2+^_in_ response was detected at the lower concentration of 1 mM glucose, exposure to 5 and 20 mM triggered a rapid twofold increase in Ca^2+^_in_ within 4 s (Fig. 5A). At 5 mM, this increase gradually declined over 120 s, whereas at 20 mM, the elevated Ca^2+^_in_ level was sustained for the remainder of the experiment, indicating a potentially new steady state with either sustained Ca^2+^ uptake or reduced Ca^2+^ efflux. In contrast, fucose elicited no significant change in Ca^2+^_in_ level under the tested conditions (see Fig. S4C at https://doi.org/10.5281/zenodo.22032718). This difference likely reflects a potential role of Ca^2+^_in_ signaling in *P. aeruginosa* recognition of a substantial supply of glucose as a carbon source.

#### Amino acids

Amino acids (AAs) are a major nutrient source and are also known to modulate *P. aeruginosa* virulence and host–pathogen interactions during infection (62). First, we examined three AAs (arginine [Arg], asparagine [Asn], and tryptophan [Trp]) previously implicated in such interactions (reviewed in reference 74) at pH 7. Challenging PAO1 cells with 1–10 mM Arg induced a significant Ca^2+^_in_ increase of up to 50% only in response to 10 mM, which peaked within 20 s (highlighted in blue) before returning to the level of the control (highlighted in green) (Fig. 5B). A significantly stronger Ca^2+^_in_ response was formed to Asn (Fig. 5C), with both 1 and 5 mM resulting in increased Ca^2+^_in_ level within 25 s, followed by a gradual return to control level over ~60 s. Trp induced a short-lived concentration-dependent increase in Ca^2+^_in_ level during the first 10 s, which subsequently returned to control level (Fig. 5D).

Next, we examined AAs with distinct chemical properties and relevance to *P. aeruginosa* pathogenicity. Methionine (Met) is a sulfur-containing AA known to modulate *P. aeruginosa* behavior*,* including inhibition of biofilm formation (75). At higher concentrations (5 and 10 mM), Met elicited a modest Ca^2+^_in_ increase, which peaked within 20 s and declined below the control levels (Fig. 5E). Glutamate (Glu), a host neurotransmitter (76) with major roles in *P. aeruginosa* nucleotide, amino acid, and cofactor biosynthesis, as well as nitrogen assimilation (reviewed in reference 77), did not elicit a detectable Ca^2+^_in_ response under the tested conditions (see Fig. S4D at https://doi.org/10.5281/zenodo.22032718).

Because AA recognition is influenced by charge, which depends on pH, we compared the Ca^2+^_in_ responses observed at pH 7 to those at pH 5 and 9 for two amino acids that showed the strongest effect but have distinct pIs: Arg (pI ~10.61) and Asn (pI ~4.71). Despite their different pIs, both AAs added at 5 mM produced similar Ca^2+^_in_ patterns: initial increase at pH 5 and 7 followed by a gradual decline and much lower increase at pH 9 followed by almost constant steady state (Fig. 5F and G). The main difference between the two AAs was a twofold stronger response to Asn at pH 5 and 7. To determine whether pH itself alters Ca^2+^ transport, we challenged PAO1 grown at 5 mM Ca^2+^ with saline at pH 5, 7, and 9 and observed no statistically significant differences in Ca^2+^_in_ (see Fig. S4E at https://doi.org/10.5281/zenodo.22032718). Similarly, PAO1 grown without Ca^2+^ and then exposed to 5 mM Ca^2+^ at pH 5–9 showed no significant differences in Ca^2+^_in_ (see Fig. S4F at https://doi.org/10.5281/zenodo.22032718). Collectively, these results indicate that while Ca^2+^ uptake itself showed no pH dependence under the conditions tested, both Ca^2+^ uptake and influx triggered by Arg and Asn were impaired at alkaline pH. This effect may be compounded by a stronger Ca^2+^ chelation by the amino acids at basic pH (78), which could limit Ca^2+^ availability.

#### Nucleotides

**Fig 6:**
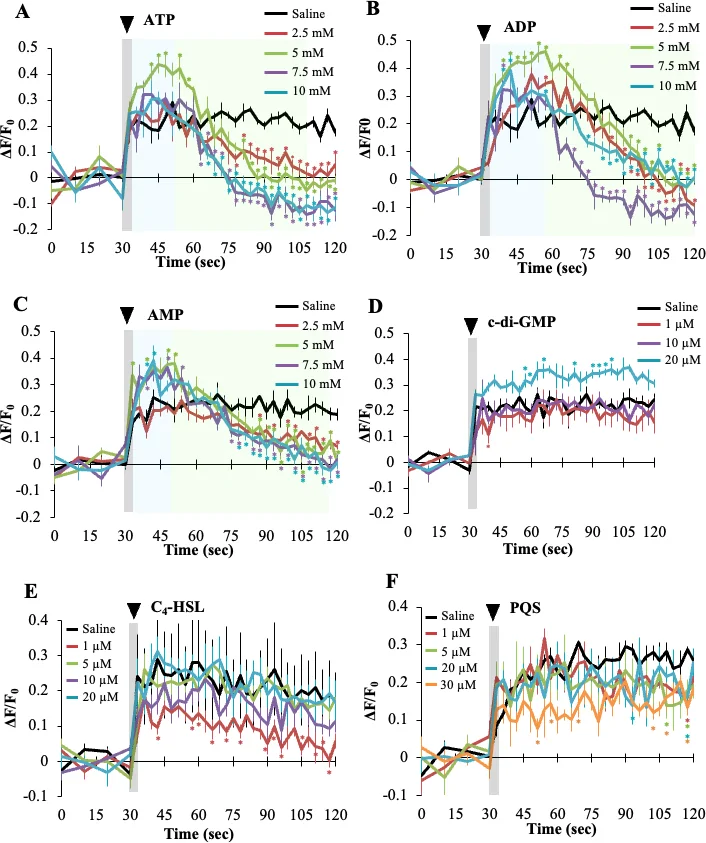
The effect of nucleotides and signaling molecules on Ca^2+^_in_ levels. Relative fluorescence change (Δ*F*/*F*_0_) of R-GECO1 in stationary-phase PAO1 grown in BMM with 5 mM Ca^2+^ was calculated as Δ*F* = RFU at each time point − *F*_0_, where *F*_0_ = average basal RFU before injection. The resulting Δ*F* was normalized by *F*_0_. ATP (**A**), ADP (**B**), AMP (**C**), c-di-GMP (**D**), C_4_-HSL (**E**), or PQS (**F**) was added at pH of 6.8–7.0 at the time indicated by the arrows, and R-GECO1 fluorescence was measured. The average relative fluorescence intensity and the standard error values obtained from four biological replicates are shown. A one-way ANOVA with Dunnett’s post hoc statistical analysis (*, *p* < 0.05) was performed.

During *P. aeruginosa* infection, lysis of both bacterial and host cells may occur and release ATP, which becomes a major signal, activating the immune response and regulating *P. aeruginosa* virulence (79–81). To test whether extracellular ATP, as well as ADP and AMP, trigger Ca^2+^_in_ response, we exposed PAO1 cells to each nucleotide (2.5–10 mM) and monitored changes in Ca^2+^_in_ levels. We observed a biphasic, concentration-dependent response (Fig. 6A through C). In the first phase (highlighted in blue), Ca^2+^_in_ level increased within 4–15 s of nucleotide addition, with the strongest effects at 5 mM ATP and ADP, and similar responses to 5–10 mM AMP. In the second phase (highlighted in green), Ca^2+^_in_ level declined either back to baseline (2.5–10 mM AMP) or below baseline (7.5–10 mM ATP and 7.5 mM ADP) (Fig. 6A through C). These data suggest that all three nucleotides induce both Ca^2+^ influx and efflux. The latter is consistent with the reported role of ATP in controlling Ca^2+^ efflux in *E. coli* (82). We also tested two nucleotide-derived signaling molecules, cyclic AMP (cAMP) and cyclic di-GMP (c-di-GMP), which are central to *P. aeruginosa* surface-associated behaviors (83–85). After the addition of these molecules (1–30 μM), we observed modest opposite impacts on Ca^2+^_in_ levels, which slightly reduced in response to 30 μM cAMP (see Fig. S5A at https://doi.org/10.5281/zenodo.22032718) but increased at 20 μM c-di-GMP (Fig. 6D), suggesting only a moderate response mostly modulating Ca^2+^ efflux.

#### Quorum-sensing signaling molecules

*P. aeruginosa* employs at least three quorum-sensing (QS) systems: LasI-LasR, RhlI-RhlR, and PQS to coordinate cell-to-cell communication and control virulence factor production during infections (86–88). These systems involve the signaling molecules N-(3-oxododecanoyl)-L-homoserine lactone (3-oxo-C_12_-HSL), N-butyryl-L-homoserine lactone (C_4_-HSL), and 2-heptyl-3-hydroxy-4(1H)-quinolone (PQS), respectively. When *P. aeruginosa* cells were exposed to 1–20 μM of C_4_-HSL, Ca^2+^_in_ levels reduced with 1 μM C_4_-HSL eliciting a more significant response and bringing Ca^2+^_in_ to the basal level in 90 s (Fig. 6E). In response to PQS, Ca^2+^_in_ levels also reduced, but a higher concentration of 30 μM showed a greater response maintaining Ca^2+^_in_ at about twofold below the control (Fig. 6F). Exposure to C_12_-HSL showed no significant change in Ca^2+^_in_ levels (see Fig. S5B at https://doi.org/10.5281/zenodo.22032718). Overall, these results indicate that two QS molecules C_4_-HSL and PQS reduce Ca^2+^_in_ but act at different concentration thresholds, likely reflecting different recognition mechanisms.

#### Oxidative stress

**Fig 7:**
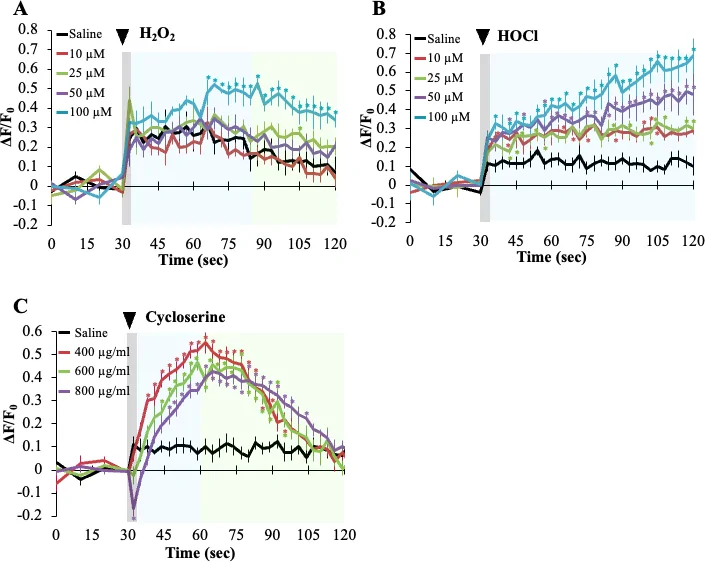
The effect of oxidative stress and cycloserine on Ca^2+^_in_ levels. Relative fluorescence change (Δ*F*/*F*_0_) of R-GECO1 in stationary-phase PAO1 grown in BMM with 5 mM Ca^2+^ was calculated as Δ*F* = RFU at each time point − *F*_0_, where *F*_0_ = average basal RFU before injection. The resulting Δ*F* was normalized by *F*_0_. H_2_O_2_ (**A**), HOCl (**B**), or cycloserine (**C**) was added at the time indicated by the arrows, and R-GECO1 fluorescence was measured. The average relative fluorescence intensity and the standard error values obtained from four biological replicates are shown. A one-way ANOVA with Dunnett’s post hoc statistical analysis (*, *p* < 0.05) was performed.

Reactive oxygen species (ROS) play a central role in killing invading pathogens during phagocytosis (89). To counteract ROS, bacterial pathogens such as *P. aeruginosa* evolved multiple defense mechanisms, including detoxifying enzymes, protein repair systems, and efflux pumps (90). To determine whether exposure to oxidative stress impacts Ca^2+^_in_, PAO1 cells were challenged with H_2_O_2_ and HOCl, two major ROS produced by neutrophils (91). Exposure to H_2_O_2_ only showed an impact at 100 μM increasing Ca^2+^_in_ mostly 30 s after the exposure followed by a gradual decrease (Fig. 7A). In contrast, HOCl induced a more pronounced and sustained Ca^2+^_in_ increase at all tested concentrations (10–100 μM), with continuous elevation observed at 50 and 100 μM (Fig. 7B). Although treatments with 100 μM of HOCl or H_2_O_2_ did not cause any leakage of R-GECO1 (see Fig. S4B at https://doi.org/10.5281/zenodo.22032718), the prolonged Ca^2+^_in_ increase may be due to oxidative stress-related cell perturbations.

#### Antibiotics

*P. aeruginosa* is becoming increasingly resistant to most, if not all, currently available antibiotics (92, 93), posing a major threat to public health. In addition, exposure to sub-minimum inhibitory concentrations (sub-MIC) of antibiotics can promote biofilm formation and enhance resistance (58–60). To examine whether *P. aeruginosa* alters Ca^2+^_in_ levels in response to antibiotics, PAO1 cells were treated with tobramycin and polymyxin B, resistance to which is enhanced by Ca^2+^ in PAO1 (29, 94), as well as cycloserine. These antibiotics have different mechanisms of action, with tobramycin interfering with translation (95), polymyxin B disrupting the outer membrane (96), and cycloserine disrupting peptidoglycan biosynthesis (97). Exposure to sub-MIC levels of tobramycin and polymyxin (29, 94) did not elicit significant changes in Ca^2+^_in_ (see Fig. S5C and D at https://doi.org/10.5281/zenodo.22032718). In contrast, cycloserine applied at sub-MIC levels (98), not causing R-GECO1 leakage (see Fig. S4B at https://doi.org/10.5281/zenodo.22032718), triggered a significant, dose-dependent Ca^2+^_in_ increase that peaked within 30 s and subsequently returned to control levels within 45–60 s (Fig. 7C). This biphasic response suggests the sequential activation of Ca^2+^ influx and efflux mechanisms and implicates Ca^2+^_in_ as a potential signaling mediator in the response to a cell wall-targeting cycloserine.

## DISCUSSION

**Fig 8:**
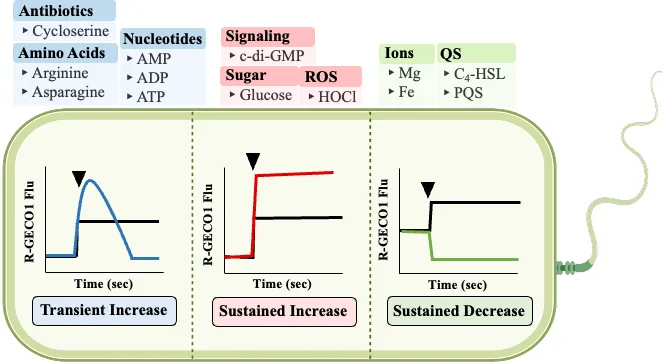
Summary of Ca^2+^_in_ responses to external stimuli. Ca^2+^_in_ in *Pa* exhibits three types of responses upon the addition of various external stimuli. When exposed to amino acids Arg, Asn, antibiotic cycloserine, and nucleotides, Ca^2+^_in_ levels undergo a transient increase representative of a conventional signaling modality. Upon exposure to high concentrations of NaCl, glucose, the signaling molecule c-di-GMP, and HOCl, a sustained increase in Ca^2+^_in_ levels was observed. While the sustained increase following exposure to 1 M NaCl is likely indicative of disturbed membrane permeability due to osmotic shock, such a response to other stimuli may result from perturbations in Ca^2+^ flux and contribute to Ca^2+^ signaling. A sustained decrease in the level of Ca^2+^_in_ detected in response to essential metals Mg and Fe, QS molecules C_4_-HSL and PQS suggests a possible relationship between Ca^2+^ efflux and essential metal homeostasis, as well as integration of other signaling networks within the cell.

The importance of Ca^2+^ signaling in bacteria is gaining attention over recent decades. Our previous studies have shown that Ca^2+^ regulates the expression of at least 30% of *P. aeruginosa* genes (19, 20, 23) and enhances the pathogen’s virulence (21, 28). The identification of Ca^2+^ sensors, such as periplasmic EfhP (19, 28, 37) and the membrane-bound BqsS/CarS (20) and LadS (35), supports the ability of *P. aeruginosa* to detect and respond to extracellular Ca^2+^. In addition, we began building evidence that Ca^2+^_in_ may play a signaling role and contribute to Ca^2+^-dependent regulation of gene expression (19, 34). Here, we applied a fluorescent probe R-GECO1 to monitor temporal changes in Ca^2+^_in_ levels in live *P. aeruginosa* cells exposed to diverse extracellular stimuli, including a range of chemically diverse host-associated molecules. These findings provide the first experimental evidence that *P. aeruginosa* alters its Ca^2+^_in_ levels in response to glucose, Arg, Asn, ATP, ADP, AMP, as well as c-di-GMP, C_4_-HSL, PQS, HOCl, and cycloserine (Fig. 8), supporting its potential as a second messenger in bacteria.

Upon entering a host, *P. aeruginosa* must recognize new cues to adapt and survive. One such cue is Ca^2+^, present in host extracellular spaces at millimolar levels. In CF, defects in the CF transmembrane conductance regulator (CFTR) lead to disrupted ion homeostasis, resulting in elevated Ca^2+^ levels in the upper airways (reviewed in reference 50), which may trigger additional adaptive responses. The observed spikes in Ca^2+^_in_ in response to 0.1–10 mM Ca^2+^_ex_ indicate that Ca^2+^-regulation of cellular responses in *P. aeruginosa* may occur not only through periplasmically located Ca^2+^ sensors but also involve a yet unidentified cytoplasmic Ca^2+^ sensor recognizing intracellular fluctuations of Ca^2+^ as a signal. In addition to elevated Ca^2+^, advanced CF is often accompanied by hypomagnesemia (99), making Mg a limited resource and creating a highly competitive environment, where both host and pathogen sequester the metal essential for survival. In *P. aeruginosa,* Mg limitation promotes biofilm formation (100) and antimicrobial resistance through regulation by the two-component system PhoPQ (101). Another target of nutritional immunity is Fe, which is also essential for both host and pathogens and is commonly sequestered and retained by the host (102, 103). However, during advanced CF, the levels of Fe^2+^ may also increase within the lungs, reaching up to ~40 µM (104). Our transcriptional studies (PRJNA874094) showed that Ca^2+^ regulates the expression of *phoPQ* and iron sequestration pathways in *P. aeruginosa* (19). The detected decrease in Ca^2+^_in_ upon exposure to both MgCl_2_ and FeSO_4_ possibly reflects activation of Ca^2+^ efflux or indicates cross-regulatory mechanisms linking Ca^2+^ signaling with Mg and Fe homeostasis, as has been recognized in plant (105) and mammalian cells (106–108).

Metabolic crosstalk between host and invading pathogens shapes the ability of bacteria to adapt within the host environment. For example, during infection, the host undergoes a substantial shift in glucose metabolism to sustain immune responses, such as activation of T cells and monocytes (109, 110). Amino acids also serve as key nutrients and signaling molecules in host–pathogen interactions and thereby influence the outcome of infection. Beyond their role as building blocks for proteins, Arg fuels nitric oxide production in macrophages, while Asn and Trp promote T-cell activation and proliferation, respectively (74). On the bacterial side, Arg promotes robust biofilm formation and reduces swarming motility (111), whereas Met inhibits *P. aeruginosa* biofilm formation, even at submicromolar (~0.5 µM) concentrations, highlighting its therapeutic potential (75). Similarly, several sugars, including glucose, fucose, and galactose, can enhance bacterial pathogenicity, yet their therapeutic inhalation has been reported to reduce bacterial attachment and lower bacterial burden *in vivo* (68–71). In this study, we observed that exposing *P. aeruginosa* cells to high levels of glucose (5–20 mM) elevated their Ca^2+^_in_ level at least twofold. Among the tested AAs, Asn elicited the most pronounced Ca^2+^_in_ response, a 2.5-fold increase at 5–10 mM, which, however, was reduced at alkaline pH. These observations align with previous reports of Ca^2+^_in_ perturbations in response to metabolites, such as butane 2,3-diol in *E. coli* (39) and flavonoid in *Rhizobium leguminosarum* (40). The effect of 10 mM glucose on Ca^2+^_in_ level has also been reported in starved *E. coli* cells, showing a similar initial increase; however, it was followed by a steep efflux of Ca^2+^_in_ (112). Together, these findings indicate a possible interplay between nutrient and Ca^2+^ signaling that is shared across diverse bacteria, highlighting the need for further research.

During infection, *P. aeruginosa* can damage host epithelium through multiple mechanisms (113), commonly leading to the release of ATP, activating immune defenses (114, 115). The extracellular ATP (eATP) may also act as a cue for the pathogen, signaling to halt twitching motility and transition to establishing infection at the site of epithelial damage (81). In this study, we observed a significant increase in Ca^2+^_in_ when cells were challenged with both ATP and ADP, with a slightly lower response to AMP. A similar Ca^2+^_in_ response to eATP is a well-established phenomenon in eukaryotic cells, where it relies on recognition by P2X/Y receptors (116, 117), which, however, have no known homologs in prokaryotes (118). Although eATP has been reported as an environmental cue that triggers changes in gene expression in *E. coli* (119), the mechanisms of eATP recognition in bacteria remain unknown. A recent study of bacterial purine sensors containing the dCache_1 domain (Cache: Calcium channels-chemotaxis) (120) identified homologs of such sensors in numerous bacterial species, including PctA in *P. aeruginosa*. This may provide a mechanistic link between eATP recognition and Ca^2+^_in_ response. It is also tempting to hypothesize that the reported regulation of gene expression by eATP is mediated by changes in Ca^2+^_in_, which will be tested in future studies.

We also tested two major signaling molecules in *P. aeruginosa*, c-di-GMP and cAMP. Levels of c-di-GMP are increased in the presence of Ca^2+^ (121) and positively regulate the transition from a free-living to a sessile biofilm mode of life (83), whereas cAMP is known to interact with transcriptional regulator vfr (122), regulating the type III secretion system (T3SS) under Ca^2+^-limiting conditions (123). Intriguingly, exposure to these molecules elicited modest but inverse changes in Ca^2+^_in_ levels, which is consistent with their different roles in cellular physiology and raises the possibility of c-di-GMP and cAMP signaling intersecting with Ca^2+^ signaling pathways.

QS is a potent intercellular communication system that regulates gene expression in response to population density, thereby facilitating bacterial adaptation. In *P. aeruginosa,* three interconnected QS systems, LasIR (C_12_-HSL), RhlIR (C_4_-HSL), and PQS, control biofilm formation, motility, and secreted factors as well as other pathogenic traits, such as iron starvation response (86, 124). Although no significant Ca^2+^_in_ response to C_12_-HSL was detected, we observed a significant reduction in Ca^2+^_in_ upon exposure to C_4_-HSL and PQS, at either low (1 µM) or higher (30 µM) levels, respectively. These findings suggest that QS signaling systems may integrate with Ca^2+^-dependent regulatory pathways during the stationary phase, which is in line with previously reported Ca^2+^ regulation of several QS-dependent processes, such as the production of extracellular proteases, secondary metabolite pyocyanin (23), and iron acquisition (19, 24).

Reactive oxygen species (ROS) are central to the innate immune response, with professional phagocytes such as neutrophils and macrophages producing ROS, including H_2_O_2_ and HOCl, to damage bacterial membranes, proteins, and DNA, ultimately killing invading pathogens (89, 125). As a highly adaptable opportunistic pathogen, *P. aeruginosa* has evolved countermeasures against oxidative stress, including detoxifying enzymes, protein repair, and chaperone systems (90). Here, we observed a significant concentration-dependent increase in Ca^2+^_in_ in response to 10–100 μM HOCl. As with osmotic shock, this increase may reflect cell envelope stress leading to enhanced Ca^2+^ uptake, with the resulting elevation in Ca^2+^_in_, which may activate additional protective mechanisms. Furthermore, the strong biphasic Ca^2+^_in_ response to cylcoserine, a D-alanine analog that interferes with cell wall synthesis in PAO1 and induces a membrane stress response (98), supports the hypothesis that Ca^2+^_in_ fluxes may act as an early signaling cue during cell envelope stress.

While this study assessed changes in pH_in_ primarily to validate the utility of the R-GECO1 probe, we observed that growth in 5 mM Ca^2+^ elevated pH_in_ in mid-log phase cells, with further increase upon subsequent Ca^2+^ exposure. These Ca^2+^-dependent pH_in_ shifts may have important physiological implications and suggest the involvement of proton-translocating ATPases, regulating pH of bacterial cytoplasm (126). Previously, we identified several P-type ATPases (MgtA/PA4825, PA3690, and KdpB/PA1634) and ATP synthase subunit AtpD/PA5554 that were transcriptionally upregulated at elevated Ca^2+^ and contributed to maintenance of Ca^2+^_in_ homeostasis (34). Further studies employing ratiometric reporters for direct quantification of [Ca^2+^]_in_ and microscopy for single-cell resolution are needed to determine which of these proteins are involved in the observed Ca^2+^-dependent pH_in_ changes in mid-log phase cells.

Overall, the findings provide the first experimental evidence that, like eukaryotes, *P. aeruginosa* alters its Ca^2+^_in_ levels in response to select, chemically diverse host-related stimuli summarized in Fig. 8. This supports the capacity of Ca^2+^_in_ as a second messenger in bacteria and lays the groundwork for the next goal of identifying the molecular mechanisms that transduce Ca^2+^_in_ signals toward downstream cellular responses.

## MATERIALS AND METHODS

### Bacterial strains and growth conditions

All strains (Table 1) used in this study are derivatives of *Pseudomonas aeruginosa* PAO1 (127), and all plasmids were constructed using *Escherichia coli* DH5α. Plasmids used in this study are listed in Table 1. *P*. *aeruginosa* strains were cultured at 37°C in Lennox L broth (LB) (128) or BMM (129). The composition of BMM was as follows (per liter of medium): sodium glutamate, 9.0 mM; glycerol, 50 mM; MgSO_4_, 0.02 mM; NaH_2_PO_4_, 0.15 mM; K_2_HPO_4_, 0.34 mM; NaCl, 145 mM; trace metal, 200 μL; and vitamin solution, 1 mL. The final pH was adjusted to 7. Trace metal mixture (per liter of 0.83 M concentrated hydrochloric acid): CuSO_4_∙5H_2_O, 5.0 g; ZnSO_4_∙7H_2_O, 5.0 g; FeSO_4_∙7H_2_O, 5.0 g; and MnCl_2_∙4H_2_O, 5.0 g. Vitamin solution (per liter of the final solution): thiamine, 0.5 g; and biotin, 1 mg. *E. coli* strains were cultivated at 37°C in Lennox LB. For plates, 1.5% (wt/vol) agar was added to the medium. Ampicillin (Ap; 100 µg/mL for *E. coli*), kanamycin (Km; 50 µg/mL), or carbenicillin (Cb; 300 µg/mL for *P. aeruginosa*) was added to the selective media.

**TABLE 1:** Bacterial strains, plasmids, and vectors used in this study^*a*^

Plasmid, strain, or primer	Relative characteristics	Source or reference
Plasmids		
pTorPE-R-GECO1	Plasmid carrying the calcium sensor R-GECO1 encoding gene, Ap^r^	Gift from Dr. M. Franklin (43)
pGP-CMV-GCaMP6f	Plasmid carrying the calcium sensor GCaMP6f encoding gene, Km^r^	Addgene plasmid #40755 (48)
pS2513·PHP	Plasmid carrying the ratiometric fluorescent indicator protein PHP (pH indicator for *Pseudomonas*) encoding gene, Km^r^	Addgene plasmid #122590 (59)
pMF36	pKK233-2 with 1.8-kb stabilization fragment for replication in *Pseudomonas*, P*_trc_* and *oriT*, Ap^r^, Cb^r^	(46)
pASK201	pMF36 with R-GECO1, Ap^r^, Cb^r^	This study
pASK203	pMF36 with GCaMP6f, Ap^r^, Cb^r^	This study
pASK204	pMF36 with PHP, Ap^r^, Cb^r^	This study
Strains		
*P. aeruginosa* PAO1	Wild-type strain	(127)
*E. coli*	F^–^, φ80d *lacZ*Δ*M15*, Δ (*lacZYA-argF*) *U169*, *endA1*, *recA1*, *hsdR17*(r_K_^–^, m_K_^+^), *deoR*, *thi-1*, *supE44*, λ, *gyrA96*, *relA1*	NEB
Primers		
XbaI-GCaMP6f-F	5′-TTTTCTAGAGGATCCATGGGTTCTCATCATCATCA-3′	
HindIII-GCaMP6f-R	5′-TTTAAGCTTTCACTTCGCTGTCATCATTTGTAC-3′	

^*a*^ Restriction sites *XbaI* and *HindIII* are underlined.

### Standard DNA manipulation

Plasmid DNA was isolated using Zyppy Plasmid Miniprep Kit (Zymo Research, Irvine, CA), and DNA was extracted from *P. aeruginosa* strains using Wizard Genomic DNA Purification Kit (Promega, Madison, WI). DNA fragments were purified from agarose gels using Zyppy Gel RNA Recovery Kit (Zymo Research). DNA concentration was determined spectrophotometrically using a NanoDrop 1000 Spectrophotometer (Thermo Scientific, Waltham, MA). Standard methods were used for transformation of competent *E. coli* cells (130), and electroporation of *P. aeruginosa* was performed as in reference 131.

### Assessment of intracellular Ca^2+^ by GECI expressing PAO1

To construct a vector carrying the red fluorescence probe R-GECO1 and replicating in *P. aeruginosa,* the probe was digested from pTorPE-R-GECO1 (43) (Addgene catalog #32465) with *Xba*I and *Hin*dIII and ligated with the similarly digested pMF36 (46) to yield pASK201. For the green fluorescence probe, GCaMP6f was amplified from pGP-CMV-GCaMP6f (48) (Addgene catalog #40755) with primers XbaI-GCaMP6f-F/HindIII-GCaMP6f-R, and, following a similar procedure, cloned into pMF36 to make pASK203. The correct plasmid sequence was confirmed by Sanger sequencing. Each vector was introduced into *P. aeruginosa* PAO1 by electroporation. Unless indicated otherwise, bacteria were cultured in 5 mL of BMM with or without 5 mM CaCl_2_ for 12 h, normalized to OD_600_ = 0.3, and 100 µL of the normalized cultures were inoculated into 100 mL of fresh BMM with or without 5 mM CaCl_2_. Cells were grown until mid-log phase (OD_600_ = 0.20 ± .03) at 37°C, 200 rpm. Then 100 µL of PAO1 carrying ASK201 or pASK203 cultures were aliquoted into a 96-well plate (Greiner Bio-One, Frickenhausen, Germany) and, when required, treated with inhibitors for 10 min. The preparation of each inhibitor (LaCl_3_, calcimycin, or vanadate) is described in our previous report (34). Fluorescence emission of R-GECO1 (λ_em_ 589 nm) or GCaMP6f (λ_em_ 513 nm) was recorded at λ_ex_ of 561 nm or 496 nm, respectively, with a Synergy Mx Multi-Mode Microplate Reader (BioTek, Winooski, VT). To measure the basal level of intracellular Ca^2+^ concentration ([Ca^2+^]_in_), the measurements were recorded for 30 s at 10-s intervals; then the cells were challenged with 10 µM to 10 mM CaCl_2_ (final concentration) using the internal Synergy injector, mixed for 1 s, and the fluorescence was recorded for 5 min at 4-s intervals. If needed, 3 mM EGTA (final concentration) was challenged 3 min after injecting Ca^2+^, mixed for 1 s, and the fluorescence was recorded for another 2 min at 4-s intervals. For normalization, Δ*F*/*F*_0_ was calculated as (*F−F*_0_)/*F*_0_, where *F*_0_ is the baseline fluorescence signal averaged and presented as the mean value of at least three independent replicates with standard error of the mean (SEM). A Student’s *t*-test was used for statistical analysis.

### Assessment of intracellular pH

To monitor intracellular pH in *P. aeruginosa,* the PHP probe (59) was cloned into pMF36. For this, PHP from pS2513·PHP (Addgene #122590) was double-digested with *Xba*I and *Spe*I and ligated with *Xba*I-digested pMF36 to yield pASK204. The direction of the inserts was confirmed using *Hin*dIII digestion. PAO1 carrying pASK204 was grown to mid-log (12 h) or stationary (24 and 36 h) phase as determined based on growth study (see Fig. S2A at https://doi.org/10.5281/zenodo.22032718). OD_600_ of the culture was recorded, and 100 µL aliquots were distributed into a 96-well plate. The fluorescence emission of PHP (λ_em_ = 515 nm) was recorded at λ_exc_ of both 405 nm and 485 nm with the Synergy Mx Multi-Mode Microplate Reader (BioTek). After a 1-min incubation, 1 mM CaCl_2_ (final concentration) was added to each well, and the fluorescence emission was recorded immediately and 10 min after the addition. The intracellular pH (pH_i_) for each sample was calculated from the *R*405/485 values obtained for the fluorescence emission plotted against the standard curve, which was generated according to the method described in reference 59 with some changes. Briefly, PAO1 cells harboring pASK204 were grown in 5 mL BMM for 12-h shaking at 37°C. The cultures were then normalized to an OD_600_ = 0.3, and a 0.1% inoculum was added to 100 mL BMM. Once cells reached mid-log or stationary phase, OD_600_ was adjusted to 0.18 or 0.3, respectively. One milliliter of the normalized culture was then pelleted by centrifugation for 5 min at 5,000 × *g*; the supernatant was removed, and the pellet was resuspended in BMM containing 20 μM CCCP with added 50 mM sodium benzoate and 50 mM methylamine HCl at pH 5.5–9.0. One hundred microliter aliquots were distributed into a black 96-well plate and incubated for 30 min. Fluorescence intensity was measured at λ emission = 515 nm, λ excitation = 315–495 nm, showing peaks at 395 nm and ~480 nm (see Fig. S2B at https://doi.org/10.5281/zenodo.22032718). The calibration curve was formed using the ratio between the excitation peaks as λexc = 395 and 480 nm (R395/480) plotted against the pH values of equilibrated cells (see Fig. S2C at https://doi.org/10.5281/zenodo.22032718). The pH_i_ value was expressed as the mean value of three independent replicates with SEM. A Student’s *t*-test was used for statistical analysis.

### Tracking changes in Ca^2+^_in_ levels upon exposure to extracellular stimuli

*P. aeruginosa* carrying pASK201 was grown in 100 mL BMM supplied with 5 mM CaCl_2_ at 37°C, 200 rpm to stationary phase (24 h and 36 h, OD_600_ ~ 0.45), and 100 µL aliquots of culture were inoculated into a 96-well plate transferred to the Synergy Mx Multi-Mode Microplate Reader. The fluorescence emission of R-GECO1 was recorded every 10 s as described above. After the initial 30 s, a stimulus of choice was added to each well by using the internal Synergy injector. Following mixing for 1 s, fluorescence emission was recorded for 10 min at 4-s intervals. The tested stimuli included (final concentration) the following: NaCl (245 µM–1 M), MgCl_2_ (10 µM–10 mM), FeSO_4_ (4 µM–1 mM), glucose (1–20 mM), Arg (1–10 mM), Asn (1–10 mM), Trp (1–10 mM), methionine (1–10 mM), ATP (2.5–10 mM), ADP (2.5–10 mM), AMP (2.5–10 mM), 2-heptyl-3-hydroxy-4(1H)-quinolone (PQS) (1–30 µM), butanol homoserine lactone (C_4_HSL) (1–30 µM), H_2_O_2_ (10–100 µM), HOCl (10–100 µM), cycloserine (400–800 µg/mL), fucose (0.1–20 mM), glutamate (0.1–10 mM), CaCl_2_ (1 mM) at pH 5–7, cAMP (1–30 µM), c-di-GMP (1–30 µM), tobramycin (0.5–4 µg/mL), polymyxin B (1–8 µg/mL), and N-(3-oxododecanoyl)-L-homoserine lactone (1–30 µM). Arg (5 mM) and Asn (5 mM), with saline as a control, were also tested at pH 5–9. All stimuli were diluted to the desired working concentration in 0.85% NaCl (saline) with no added Ca^2+^. All experiments were done with at least three biological replicates and repeated to ensure consistency.

To determine the amount of Fe^2+^ remaining after fluorescence measurements, a ferrozine-based assay was used as previously described (132). Briefly, samples were added to a clear 96-well microplate and incubated for 30 min at room temperature. Ferrozine reagent (final 0.5 mM) was then added, followed by a 10-min incubation, and absorbance was measured at 562 nm using a Synergy Microplate Reader. Under the experimental conditions, at least 70% of the initially added Fe^2+^ remained.

To assess whether selected treatments caused membrane disturbance, RGECO-1 fluorescence was measured in PAO1 supernatants collected upon exposure. For this, PAO1 carrying pASK201 was grown in BMM as described above, collected, treated with the respective stimuli for 1 min, and centrifuged at 11,600 × *g* for 1 min. Both pellets and supernatants were collected, and the pellet was resuspended in BMM containing 5 mM Ca^2+^. The samples were then added to a black 96-well plate at a volume of 120 µL. RGECO-1 fluorescence was recorded along with OD_600_ (for the pellet). The tested stimuli included (final concentration) the following: NaCl (145 mM [saline] and 1 M), HOCl (100 µM), H_2_O_2_ (100 µM), and cycloserine (600 and 800 µg/mL). All stimuli were diluted to the desired working concentration in saline with no added Ca^2+^. All the experiments were done with at least four biological replicates.

### Statistical analysis

When applicable, a Student’s *t*-test (Fig. 1B and C), a one-way ANOVA followed by Tukey-Kramer post hoc test (for all pairwise comparisons), or one-way ANOVA followed by Dunnett’s post hoc test (for many-to-one control comparisons) was performed to evaluate statistical significance. These analyses were conducted in R (version 4.5.2) using RStudio (version 2025.09.2 + 418). Three to four independent biological replicates were included in each experiment. Values of *p* < 0.05 were considered significant and depicted as a dot above the respective time point or as an asterisk as detailed in the figure legends.
